# The RNA-binding protein RBM24 regulates lipid metabolism and SLC7A11 mRNA stability to modulate ferroptosis and inflammatory response

**DOI:** 10.3389/fcell.2022.1008576

**Published:** 2022-11-21

**Authors:** Jin Zhang, Xiangmudong Kong, Wenqiang Sun, Leyi Wang, Tong Shen, Mingyi Chen, Xinbin Chen

**Affiliations:** ^1^ Comparative Oncology Laboratory, Schools of Veterinary Medicine and Medicine, UC, Davis, CA, United States; ^2^ West Coast Metabolomics Center, UC, Davis, CA, United States; ^3^ Department of Pathology, Southwestern Medical Center, University of Texas, Austin, TX, United States

**Keywords:** Rbm24, Rbm38, SLC7A11, ferroptosis, inflammatory response, mRNA stability, liver steatosis

## Abstract

Lipids play a critical role in many cellular processes by serving as structural components of cell membranes or functioning as energy fuel and signaling molecules. The RNA-binding proteins RBM24 and RBM38 share an identical RNA-binding domain and thereby, regulate a group of same targets, such as p21. However, it is not certain whether RBM24 and RBM38 participates in lipid homeostasis. Here, lipidomic analysis showed that a deficiency in RBM24 or RBM38 leads to altered lipid metabolism, with more profound alteration by loss of RBM24 in MCF7 cells. We also showed that mice deficient in RBM24 were prone to chronic inflammation and liver steatosis, but not spontaneous tumors. These data let us speculate whether RBM24 regulates ferroptosis, a programmed cell death that links inflammation and liver steatosis *via* lipid peroxidation. Indeed, we found that over-expression of RBM24 protected, whereas knockout of RBM24 sensitized, cells to Erastin-induced ferroptosis by modulating the mRNA stability of SLC7A11, a ferroptosis inhibitor. Moreover, we showed that knockdown of SLC7A11 reversed the effect of RBM24 on ferroptosis. Together, our study revealed that RBM24 regulates lipid metabolism and SLC7A11 mRNA stability to modulate ferroptosis and inflammatory response.

## Introduction

RNA-binding proteins (RBPs) are key regulators of post-transcriptional processes such as alternative splicing, mRNA stability, polyadenylation and mRNA localization (Gebauer et al., 2021). RBM24 is an RNA-binding protein that plays a critical role in multifaceted cellular processes, including cell differentiation and cell cycle regulation. RBM24 contains one RNA Recognition Motif (RRM) and is preferentially expressed in muscle tissues (Yang et al., 2014). Importantly, studies from our and other groups showed that Rbm24-null mice are embryonic lethal due to defects in the heart development (Yang et al., 2014; Zhang et al., 2018a). Mechanistically, RBM24 was found to mediate muscle-specific exon inclusion for several mRNAs that are required for muscle cell differentiation (Yang et al., 2014; Lu et al., 2022). In addition to modulate alternative splicing, RBM24 was found to modulate the stabilities of CDKN1A mRNA *via* binding to an AU-rich element and thereby, regulates cardiomyocytes differentiation (Jiang et al., 2014). Recently, RBM24 was also found to modulate mRNA translation of crystallin mRNAs in the lens *via* cytoplasmic polyadenylation (Shao et al., 2020a), indicating a critical role of RBM24 in lens development. Nevertheless, it is still unclear whether RBM24 is involved in other physiological process.

Previously studies from our group and others indicated that RBM24 contains an identical RNA-binding domain as RNA-binding protein RBM38 and thereby, regulate a same group of targets, including p63 and p21, *via* mRNA stabilities and p53 *via* mRNA translation (Shu et al., 2006; Zhang et al., 2010; Zhang et al., 2011). We also found that a motif at the C-terminus of RBM24 is conserved between RBM24 and RBM38 (Jiang et al., 2014), which mediates the interaction of RBM24 and RBM38 with eukaryote initiation factor 4E (eIF4E) and prevents it from binding to the 5′-cap of p53 mRNAs (Zhang et al., 2018a). Subsequent studies also showed that the interaction between RBM24/38 with eIF4E can be disrupted by a small peptide (Zhang et al., 2018a; Lucchesi et al., 2019). However, the amino acid sequences of RBM24 and RBM38 are quite different outside the RNA-binding domain and C-terminus, suggesting that RBM24 and RBM38 cooperate with different binding partners and thereby have their own distinct functions. Indeed, previous studies have shown that RBM24 and RBM38 are involved in different physiological processes. For example, RBM24 is known to be required for heart development (Yang et al., 2014; Zhang et al., 2018a), whereas RBM38 was found to regulate hematopoiesis but is dispensable for health heart function (van den Hoogenhof et al., 2017; Zhang et al., 2014). Interestingly, previous studies showed that Rbm38 is frequently altered in various types of cancers and Rbm38-deficient mice were prone to spontaneous tumors (Zhang et al., 2014; Zou et al., 2021). However, whether RBM24 plays a role in tumorigenesis remains unclear.

Lipids represent a complex group of biomolecules, including cholesterol and cholesterol esters (CEs), triglycerides (TAGs) and phospholipids. Lipids are required for the maintenance of cellular structures, energy supply, and diverse aspects of signal transduction (Snaebjornsson et al., 2020). Studies have shown that cancer cells exhibit specific alterations in lipid homeostasis, such as lipogenesis, lipid uptake and storage (Snaebjornsson et al., 2020; Broadfield et al., 2021; Hoy et al., 2021). In addition, many disease conditions such as obesity, chronic inflammation and nonalcoholic fatty liver disease (NAFLD), are also known to be resulted from altered lipid metabolism (Singla et al., 2010; Carotti et al., 2020; Pei et al., 2020; Hoy et al., 2021).

To understand the physiological function of RBM24, we hypothesize that RBM24 plays a role in modulating lipid metabolism. To this end, lipidomic analysis was performed with RBM24-and RBM38-KO cells. Interestingly, we found that loss of RBM24 leads to a distinct change in several lipid species, including diglycerides, triglycerides, and phospholipids. Next, to further understand the biological functions of RBM24, we generated a cohort of Rbm24-deficint mice and monitored them throughout their lifespan for pathological abnormalities. Interestingly, we found that Rbm24-deficiency led to increased liver steatosis and chronic inflammation but not spontaneous tumors in mice. To uncover the underlying mechanism of the alterations mediated by RBM24-deficiency, we focused on the role of RBM24 in regulating ferroptosis, a programmed cell death that links lipid metabolism, chronic inflammation and liver steatosis. Indeed, we found that RBM24 protect cancer cells from ferroptosis by stabilizing mRNA of SLC7A11, an inhibitor of ferroptosis. Together, our study revealed that RBM24 regulates lipid metabolism and SLC7A11 mRNA stability to modulate ferroptosis and inflammatory response.

## Materials and methods

### Reagents

Scrambled siRNA (5′- GGC CGA UUG UCA AAU AAU U -3′) and SLC7A11 siRNA (5′-CCA GAU AUG CAU CGU CCU U-3′ and 5′-AAU GUG GCC UAC UUU ACG A-3′) were purchased from Dharmacon (Chicago, IL). For siRNA transfection, RNAiMax (Life Technologies) was used according to the user’s manual. Proteinase inhibitor cocktail, Actinomycin D, Erastin were purchased from Sigma-Aldrich (St. Louis, MO). Magnetic Protein A/G beads were purchased from MedChem (Santa Clara, CA). RiboLock RNase Inhibitor and Revert Aid First Strand cDNA Synthesis Kit were purchased from Thermo Fisher (Waltham, MA). WesternBright ECL HRP substrate was purchased from Advansta (San Jose, CA).

### Mice

*Rbm24*-deficient mice were generated by UC Davis Mouse Biology Program as described previously (Zhang et al., 2018a). Rbm38-deficient mice were generated by UC Davis Mouse Biology Program as previously described (Zhang et al., 2014; Zhang et al., 2018b). This study included 56 of wil-type mice (23 females and 33 males), 30 of *Rbm38* ^*−/−*^ mice (11 females and 19 males) and 22 of *Rbm24* ^*+/−*^ mice (9 females and 13 males). All the mice were mornitored throughout their lifespan. A mouse was euthanized when it developed signs of sickness or weight loss or failure to eat or drink. Once used, all the major organs from a mouse, such as lung, spleen, liver, stomach, pancreas, salivary gland, GI-tract were collected for histological analysis. All animals and use protocols were approved by the University of California at Davis Institutional Animal Care and Use Committee.

### Cell culture, cell line generation, and siRNA knockdown

MCF7, HCT116, and p53^−/−^ HCT116 cells and their derivatives were cultured in DMEM (Dulbecco’s modified Eagle’s medium, Invitrogen) supplemented with 10% fetal bovine serum (Hyclone). p53^−/−^HCT116 cells that can inducibly expressing ectopic RBM24 were generated as previously described (Jiang et al., 2014). RBM24-KO MCF7 and HCT116 were generated previously by using CRISPR-Cas9 technology (Zhang et al., 2018a). To knock down SLC7A11, cells were transiently transfected with a scrambled or SLC7A11 siRNA for 3 days using RNAiMax (Life Technologies) according to user’s manual.

### Western blot analysis

Western blot analysis was performed as previously described (Dohn et al., 2001). Briefly, Cells were washed with cold PBS, pelleted, and resuspended 2×SDS sample buffer. Proteins were separated in an SDS-polyacrylamide gel and transferred to a nitrocellulose membrane, followed by incubation with primary and secondary antibodies. The membrane was then incubated with ECL substrate to visualize the expression of a protein by using UVP Chemistudio from Analytik Jena (Upland, CA). The antibodies used in this study were: anti-Actin (Santa Cruz Biotechnology, sc-47778, 1:3000); anti-SLC7A11 (Cell signaling, Cat# 12691, 1:2000); anti-vinculin (Santa Cruz Biotechnology, Cat# sc-73614, 1:3000); and anti-HA (Covance, Cat# 901513, 1:2000); anti-RBM24 was generated as previously described (Zhang et al., 2018a).

### RNA isolation and RT-PCR

Total RNA was isolated with Trizol reagent as described according to user’s manual, followed by cDNA synthesis using Revert Aid First Strand cDNA Synthesis Kit. The PCR program used was (i) 94°C for 4 min, (ii) 94°C for 30 s, (iii) 60°C for 30 s, (iv) 72°C for 45 s, and (v) 72 °C for 10 min. From steps 2 to 4, the cycle was repeated 22 times for actin, 28–35 times depending on the targets. The primers for the actin were forward primer 5′- TCC ATC ATG AAG TGT GAC GT-3′ and reverse primer 5′-TGA TCC ACA TCT GCT GGA AG-3’. The primers for SLC7A11 were a forward primer, 5′-CTC CTG CTT TGG CTC CAT GA-3′, and a reverse primer, 5′-GGA CGA TGC ATA TCT GGG CA-3’. The primers for the human TFRC were a forward primer 5′- GAG GAG CCA GGA GAG GAC TT-3′ and a reverse primer 5′- ACG CCA GAC TTT GCT GAG TT-3’. The primers for the human LPCAT4 were a forward primer 5′- GCC GGT CTT AGT GAG GAG CAG CTT C-3′ and a reverse primer 5′- ACG GAA AGG TTC TCA GCT CGG GAC-3’.

### mRNA stability assay

mRNA stability assay was performed as previously described (Scoumanne et al., 2011). Cells were mock-treated or treated with Actinomycin D (10μg/ml) for various time, followed by total RNA isolation. The total RNAs were used for cDNA synthesis, followed by PCR amplification with actin and SLC7A11 primers. The relative percentage of remaining SLC7A11 RNA was calculated by normalizing SLC7A11 mRNA from triplicate samples (mean ± SD) and then plotted to calculate the half-life of SLC7A11 mRNA.

### RNA-chip assay

RNA Chip analysis was performed as previously described (Peritz et al., 2006). Briefly, cells were lysed with polysomal lysis buffer and cell lysates were then immunoprecipitated with 1 μg of anti-HA or mouse IgG at 4°C overnight. The RNA-protein immunocomplexes were precipitated by protein A/G beads and subjected to RT-PCR.

### Lipidomic LC-MS/MS analysis

Lipids were extracted and analyzed by reversed-phase liquid chromatography tandem mass spectrometry (RPLC-MS/MS) at West Coast Metabolomics Center with published methods (Ding et al., 2021). Briefly, 30 million cells were harvested and homogenized in 225 µL of cold methanol which contained class-representative deuterated lipid internal standards. Lipids were extracted with the Matyash method (Matyash et al., 2008). 750 µL of methyl tertiary-butyl ether (MTBE) was added, followed by 188 µL of water. Samples were vortexed, shaken at 4 °C, and then centrifuged. The top non-polar layer was transferred to a new tube and dried down. Each lipid extract was resuspended using a mixture of methanol/toluene (9:1, v/v) (110 µL) containing a synthetic standard 12-[(cyclohexylamino) carbonyl]amino]-dodecanoic acid (CUDA) as quality control. Method blanks were extracted from empty tubes for subsequent blank subtraction. Aliquots from every lipid extract were pooled as matrix-representative quality control (PoolQC). Extracted lipids were injected into a Vanquish UHPLC system (Thermo Scientific) coupled with a Q-Exactive HF Orbitrap MS instrument (Thermo Scientific). 2 µL was injected for both positive and negative ionization mode analyses. The liquid chromatography was using a Waters Acquity UPLC CSH C18 column (100 × 2.1 mm; 1.7 µm). The mobile phases for positive mode were (A) 60:40 acetonitrile:water with 10 mM ammonium formate and 0.1% formic acid and (B) 90:10 isopropanol:acetonitrile with 10 mM ammonium formate and 0.1% formic acid; for negative mode, the mobile phase modifier was 10 mM ammonium acetate instead. The column temperature was 65°C. The gradient ramped from 15% mobile phase B to 99% over 12 min, followed by 2 min of re-equilibration. Top4 data-dependent MS/MS acquisition was exploited for the untargeted lipidomics. The parameters were as the following: mass range 120–1700 m/z; spray voltage 3.6 kV (ESI+) and −3 kV (ESI−), sheath gas flow rate 60 units; auxiliary gas flow rate 25 units, capillary temperature 320°C, full scan MS1 mass resolving power 60,000 scan, dd-MS/MS mass resolving power 15,000, normalized collision energy at 20%, 30%, and 40%. Thermo Xcalibur™ 4.0.27.19 was used for data acquisition. Data processing and annotation were conducted in MS-DIAL 4.18 (Tsugawa et al., 2020) with these parameters: MS1 tolerance is 8 mDa, MS2 tolerance 10 mDa, minimum peak height 50,000 counts, smoothing level 3 scans, minimum peak width 10 scans, considered adducts [M + H]^+^ [M + NH4]^+^ [M + Na]^+^ [M + K]^+^ [M-H]^-^, and [M + CH3COO]^-^. Lipids were annotated by matching accurate precursor masses, retention times, and MS/MS spectra against MS-DIAL built-in lipid library and in-house library with a confidence score >70%. Data were further processed with MS-FLO to merge adducts and remove duplicated features (DeFelice et al., 2017). Data were normalized to the total amount of identified lipids to correct for instrumental signal drift. The signals with high variance (RSD >30% in PoolQC) and background ions (in method blanks) were removed.

### Histology analyses

Mouse tissues from major organs were fixed in neutral buffered formalin for 18 h and embedded in paraffin blocks. Tissue blocks were sectioned (6 μm) and stained with hematoxylin and eosin.

### Statistical analysis

The Log-rank test was used for Kaplan–Meier survival analysis. Fisher’s exact test was performed for the statistical analysis. Values of *p* < 0.05 were considered significant.

### Cell viability assay

Cell viability was measured using CellTiter-Glo^®^ kit (Promega) according to manufacturer’s guidelines. Briefly, 5 × 10^3^ cells were seeded in a well of a 96-well plate in triplicates, followed by mock-treatment or treatment with Erastin for 8 h. The ATP content was measured by adding CellTiter-Glo^®^ reagent and recording luminescence using Luminometer (SpectraMAX) after a 10 min equilibration period. Data were shown in mean ± SD (n = 3).

## Results

### A deficiency in RBM24 or RBM38 leads to distinct alterations in lipid profile

**FIGURE 1:**
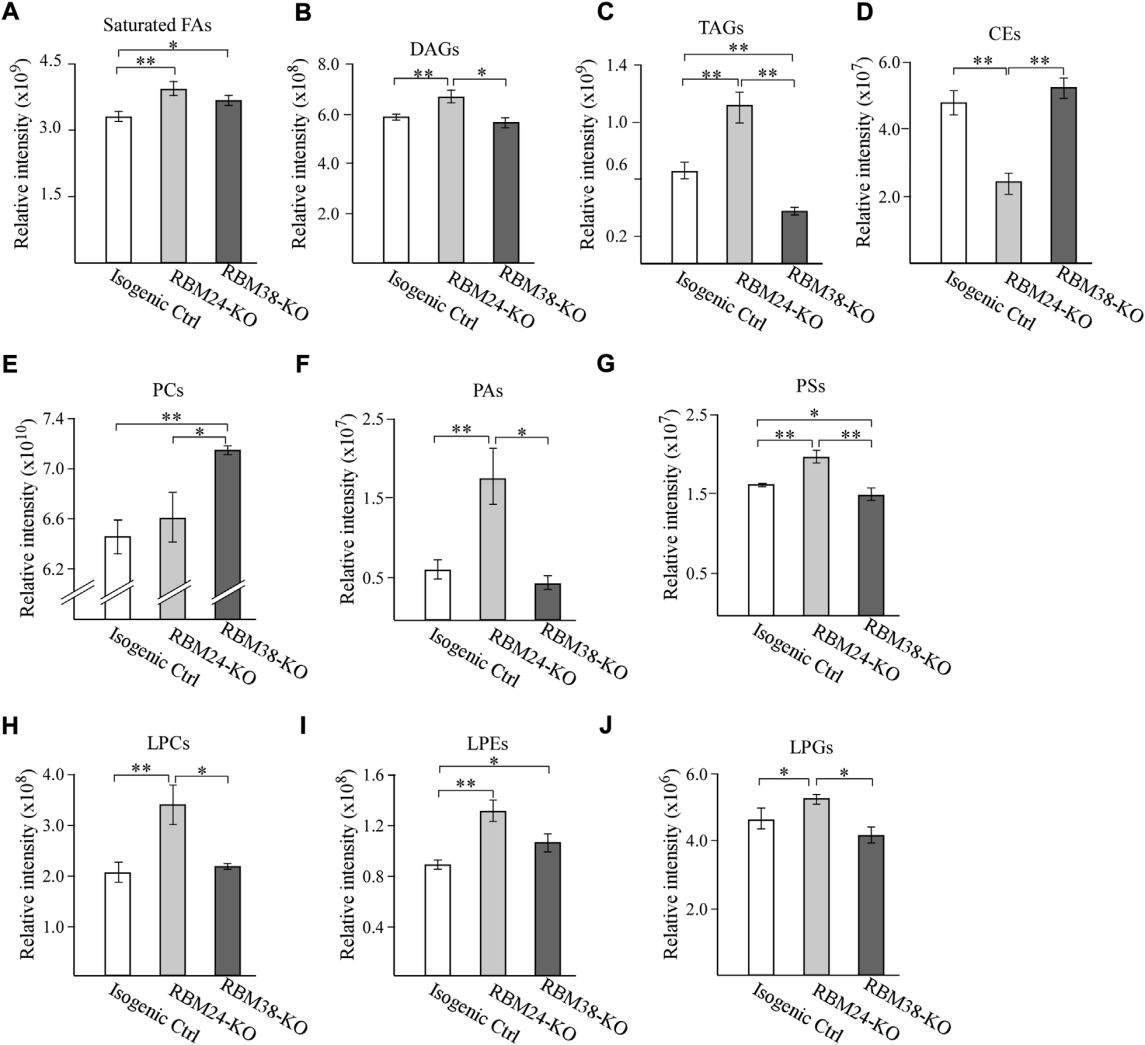
A deficiency in *RBM24* or *RBM38* leads to distinct alterations in lipid profile **(A–J)** Isogenic control, *RBM24* ^*−/−*^, and *RBM38* ^*−/−*^ MCF7 cells were used for lipidomic LC-MS/MS analysis. The relative abundance of a lipid was calculated as mean ± SEM and statistical significance was determined using Student’s t-test. **(A)**: Saturated Fatty Acids **(B)**: Diacylglycerols (DAGs) **(C)**: Triglycerides (TAGs) **(D)**: Cholesterol esters (CEs) **(E)**: phosphatidylcholines (PCs) **(F)**: phosphatidic acids (PAs) **(G)**: phosphatidylserines (PSs) **(H)**: lysophosphatidylcholines (LPCs) **(I)**: lysophosphatidylethanolamines (LPEs) **(J)**: lysophosphatidylglycerols (LPGs).

To examine whether RBM24 and RBM38 regulates lipid metabolism, untargeted lipidomic analyses were performed with isogenic control, Rbm24-KO and RBM38-KO MCF7 cells. Total 795 lipids were annotated through LC-MS/MS analysis, including fatty acids, triglycerides (TAGs), diglycerides (DAGs), cholesterol esters (CEs) and various phospholipids and lysophospholipids. We found that when comparing with isogenic controls, loss of RBM24 leads to a significant increase in the content of saturated fatty acids, DAGs and TAGs but a marked decrease in CEs (Figures 1A–D). By contrast, RBM38-KO cells showed a marked increase in saturated fatty acids but a decrease in TAGs when compared to isogenic controls (Figures 1A–D). Interestingly, RBM24-KO cells even showed significant increase in DAGs and TAGs but a marked decrease in CEs when compared to RBM38-KO cells (Figures 1A–D).

RBM24-or -RBM38 deficiency also leads to alteration of various phospholipids and lysophospholipid, which are the major membrane lipids for lipid bilayers. For phospholipids, we observed that when compared to isogenic control cells, loss of Rbm24 led to a significant increase in phosphatidic acids (PAs) and phosphatidylserine (PSs) but no alteration in phosphatidylcholine (PCs) (Figures 1E–G). By contrast, RBM38-KO led to marked increase in PCs, no difference in PAs, and decrease in PSs when compared to isogenic control cells (Figures 1E–G). Additionally, RBM24-KO cells showed marked decrease in PCs but increased in PAs and PSs when compared to RBM38-KO cells. For lysophospholipids, we found that when compared to isogenic control cells, RBM24-KO led to marked increase in lysophosphatidylcholines (LPCs), lysophosphatidylethanolamine (LPEs) and lysophosphatidylglycero (LPGs) whereas RBM38-KO only led to increase in LPEs (Figures 1H–J). In addition, the increase in LPCs and LPGs by RBM24-KO was also significantly higher than that by RBM38-KO (Figures 1H–J). Together, these data indicate that RBM24-or RBM38-KO leads to altered lipid metabolism with more profound changes by RBM24-KO.

### Mice deficient in *Rbm24* were prone to chronic inflammation and liver steatosis

**FIGURE 2:**
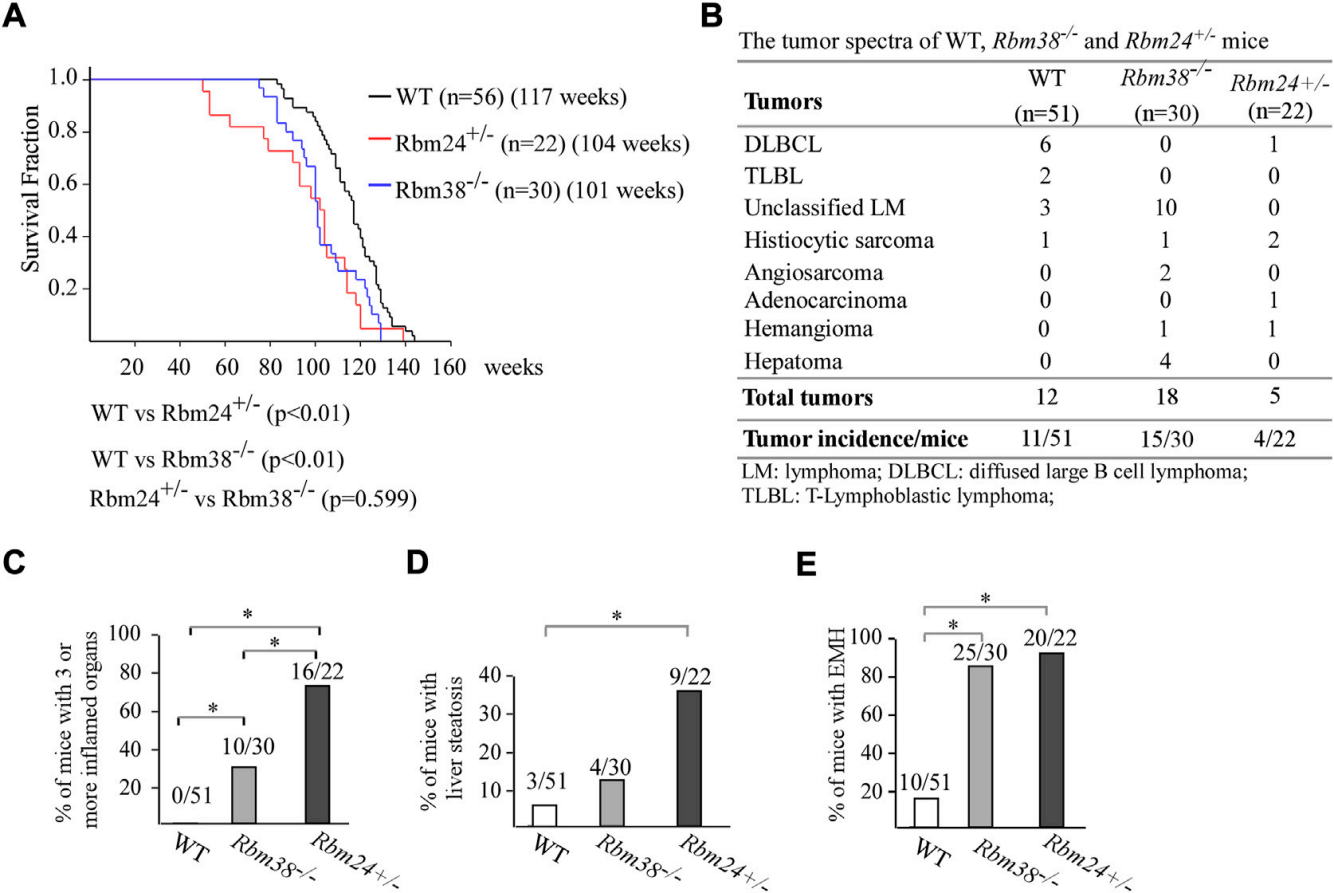
Mice deficient in *Rbm24* were prone to chronic inflammation and liver steatosis. **(A)** Kaplan-Meyer survival curves of WT (N = 56), *RBM24* ^*+/−*^ (N = 22), AND *RBM38* ^*−/−*^ (N = 30) MICE. *p*-value WAS CALCULATED BY LogRank Test. **(B)** Tumor spectra in WT (n = 51), *Rbm24* ^*+/−*^ (n = 22), and *Rbm38* ^*−/−*^ (n = 30) mice. *p* = 0.76 (WT vs. *Rbm24* ^*+/−*^); *p* = 0.0131 (WT vs. *Rbm38* ^*−/−*^); *p* = 0.0227 (*Rbm24* ^*+/-*^vs *Rbm38* ^*−/−*^) by Fisher’s exact test **(C)** Percentage of WT (n = 51), *Rbm24* ^*+/−*^ (n = 22), and *Rbm38* ^*−/−*^ (n = 30) mice with 3 or more inflamed organs. * indicated *p* < 0.05 by Fisher’s exact test. **(D)** Percentage of WT (n = 51), *Rbm24* ^*+/−*^ (n = 22), and *Rbm38* ^*−/−*^ (n = 30) mice with liver steatosis. * indicated *p* < 0.05 by Fisher’s exact test. **(E)** Percentage of WT (n = 51), *Rbm24* ^*+/−*^ (n = 22), and *Rbm38* ^*−/−*^ (n = 30)mice with extramedullary hematopoiesis (EMH). * indicated *p* < 0.05 by Fisher’s exact test.

Altered lipid metabolism is known to cause many diseases. Thus, to understand the biological function of RBM24, a cohort of WT, *Rbm24* ^*+/−*^, and *Rbm38* ^*−/−*^ mice were generated and monitored for potential abnormalities throughout their lifespan. We would like to note that both WT and *Rbm38* ^*−/−*^ mice have been generated and reported previously (Zhang et al., 2014; Zhang et al., 2017; Sun et al., 2021). The median lifespan for *Rbm24* ^*+/−*^ mice was 104 weeks, which was significantly shorter than 117 weeks for WT mice (Figure 2A). There was no difference in the lifespan between *Rbm24* ^*+/−*^ and *Rbm38* ^*−/−*^ mice (Figure 2A). Next, histopathological analyses were performed to examine the potential pathological abnormalities in these mice. We found that 11 out of 51 WT mice, 15 out of 30 Rbm38^−/−^ mice, and 4 out of 22 *Rbm24* ^*+/−*^ mice developed spontaneous tumors (Figure 2B, Supplementary Tables S1-3). Statistical analyses indicted that the tumor incidence for *Rbm24* ^*+/−*^ mice was not significantly different from that for WT mice (*p* = 1.0 by Fisher’s exact test), but less than *Rbm38* ^*−/−*^ mice (*p* = 0.0227 by Fisher’s exact test) (Figure 2B). Moreover, we found that mice deficient in Rbm24 were prone to systemic inflammation (Supplemental Table S3 and Supplementary Figure S1A). To assess inflammation, we compared the percentage of mice that developed inflammation in 3 or more organs. We found that 0 out of 51 WT mice, 10 out of 30 *Rbm38* ^*−/−*^ mice, and 16 out of 22 *Rbm24* ^*+/−*^ mice showed inflammation in more than 3 organs (Figure 2C, Supplementary Tables S1-3). Statistical analyses showed that the incidence of chronic inflammation was significantly higher in *Rbm24* ^*+/−*^ mice than that in *Rbm38* ^*−/−*^ or WT mice (*Rbm24* ^*+/−*^ vs *WT*: *p* = 0.0001, *Rbm24* ^*+/−*^ vs *Rbm38* ^*−/−*^: *p* = 0.0107, by Fisher exact test). In addition to inflamamtion, Rbm24^+/−^mice were prone to liver steatosis (Supplemental Table S3 and Supplementary Figure S1B). We found that 3 out of 51 WT, 4 out of 30 *Rbm38* ^*−/−*^ mice, and 9 out 22 *Rbm24* ^*+/−*^ mice developed liver steatosis (Figure 2D). The incidence of liver steatosis was significantly higher in *Rbm24* ^*+/−*^ mice than that in WT mice (*p* = 0.0006 by Fisher’s exact test). However, *Rbm38* ^*−/−*^ mice showed no difference in liver steatosis when comparing to either WT and *Rbm24* ^*+/−*^ mice (Figure 2D). Furhtermore, we found that like *Rbm38* ^*−/−*^ mice (25 out of 30), *Rbm24*-deficient mice (20 out of 22) were prone to extramedullary hematopoiesis (EMH) when compared to WT mice (10 out 51) (Figure 2E). Together, these data indicated that Rbm24-deficient mice had a shortened lifespan and developed distinct phenotypes when comparing to *Rbm38*-deficient mice.

### RBM24 protects cell from ferroptosis

**FIGURE 3:**
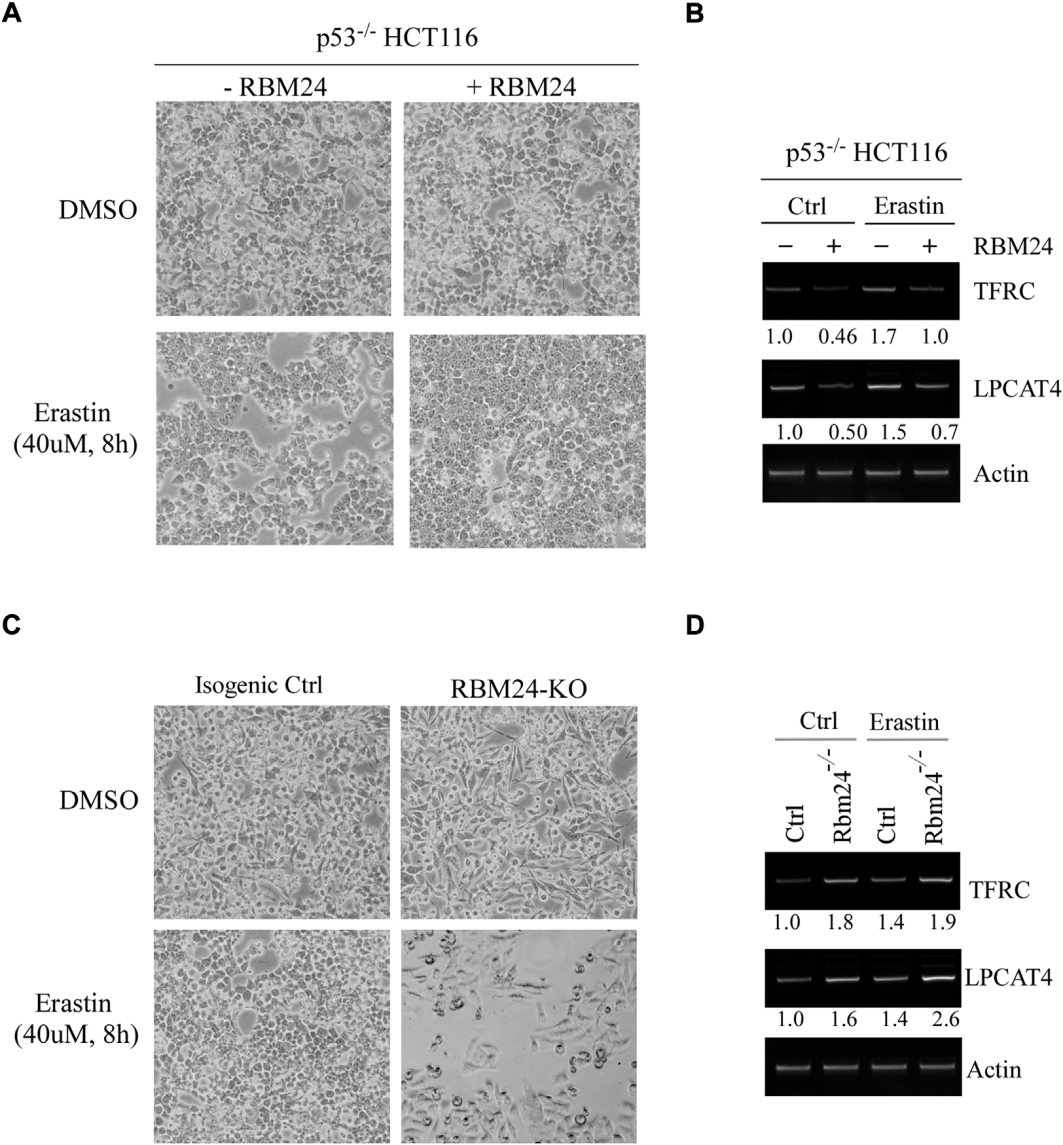
RBM24 protects p53^−/−^ HCT116 cells from ferroptosis. **(A)** p53^−/−^ HCT116 cells were uninduced or induced to express RBM24 for 24 h, followed by mock treatment or treatment with Erastin (40 uM) for 8 h. Representative microscopic pictures were taken at the endpoint. **(B)** The levels of TFRC, LPCAT4, and actin mRNAs were measured in p53^−/−^ HCT116 cells treated as in **(A)**. The relative mRNA abundance was shown below each lane. **(C)** Isogenic control and RBM24-KO HCT116 cells were mock-treated or treated with Erastin (40 μM) for 8 h, followed by microscopic imaging. **(D)** The levels of TFRC, LPCAT4, and actin mRNAs were measured in cells treated as in **(C)**. The relative mRNA abundance was shown below each lane.

Several studies have shown that lipid peroxidation would induce a programmed cell death, called ferroptosis, which would lead to the release of inflammatory cytokines, defined as danger-associated molecular patterns (DAMPs), and subsequently promote inflammation (Anderton et al., 2020; Liang et al., 2022). Interestingly, ferroptosis has been shown to be closely associated with both liver steatosis and chronic inflammation (Tsurusaki et al., 2019; Wu et al., 2021). Since RBM24 deficiency led to increased incidence in both liver steatosis and chronic inflammation (Figures 2C,D), we thus hypothesized that RBM24 regulates ferroptosis. We would like to note that since liver steatosis and chronic inflammation was less prominent in RBM38-deficient mice, the role of RBM38 in ferroptosis was not investigated in this study. To test this, p53^−/−^ HCT116 cells that can inducibly express RBM24 were mock-treated or treated with Erastin, a potent and selective inhibitor of SLC7A11 that can induce ferroptosis (Dixon et al., 2012; Dixon et al., 2014). We would like to note that p53^−/−^ HCT116 cells were used to rule out the effect of p53 since SLC7A11 was found to be repressed by p53 (Jiang et al., 2015). We found that under a mock-treatment condition, cell growth was unaltered regardless of RBM24 expression (Figure 3A). Notably, in response to Erastin treatment, expression of RBM24 in p53^−/−^ HCT116 cells inhibited Erastin-induced ferroptosis (Figure 3A). To verify this, we measured two biomarkers of ferroptosis (Proneth and Conrad, 2019; Chen et al., 2021): FTRC (transferritin receptor), which mediates uptake of circulating iron, and LPCAT4 (Lysophosphatidylcholine Acyltransferase 4), which converts lyso-phospholipids to phospholipids. We found that the levels of FTRC and LPCAT4 mRNA were markedly inhibited by RBM24 under mock and erastin-treated conditions (Figure 3B). To verify this, the same experiment was performed with isogenic control and RBM24-KO HCT116 cells. We found that knockout of RBM24 sensitized HCT116 cells to Erastin treatment along with enhanced expression of these ferroptosis markers (Figures 3C,D). Together, these data indicate that RBM24 protects cells from ferroptosis.

### RBM24 inhibits ferroptosis *via* modulating SLC7A11 expression

**FIGURE 4:**
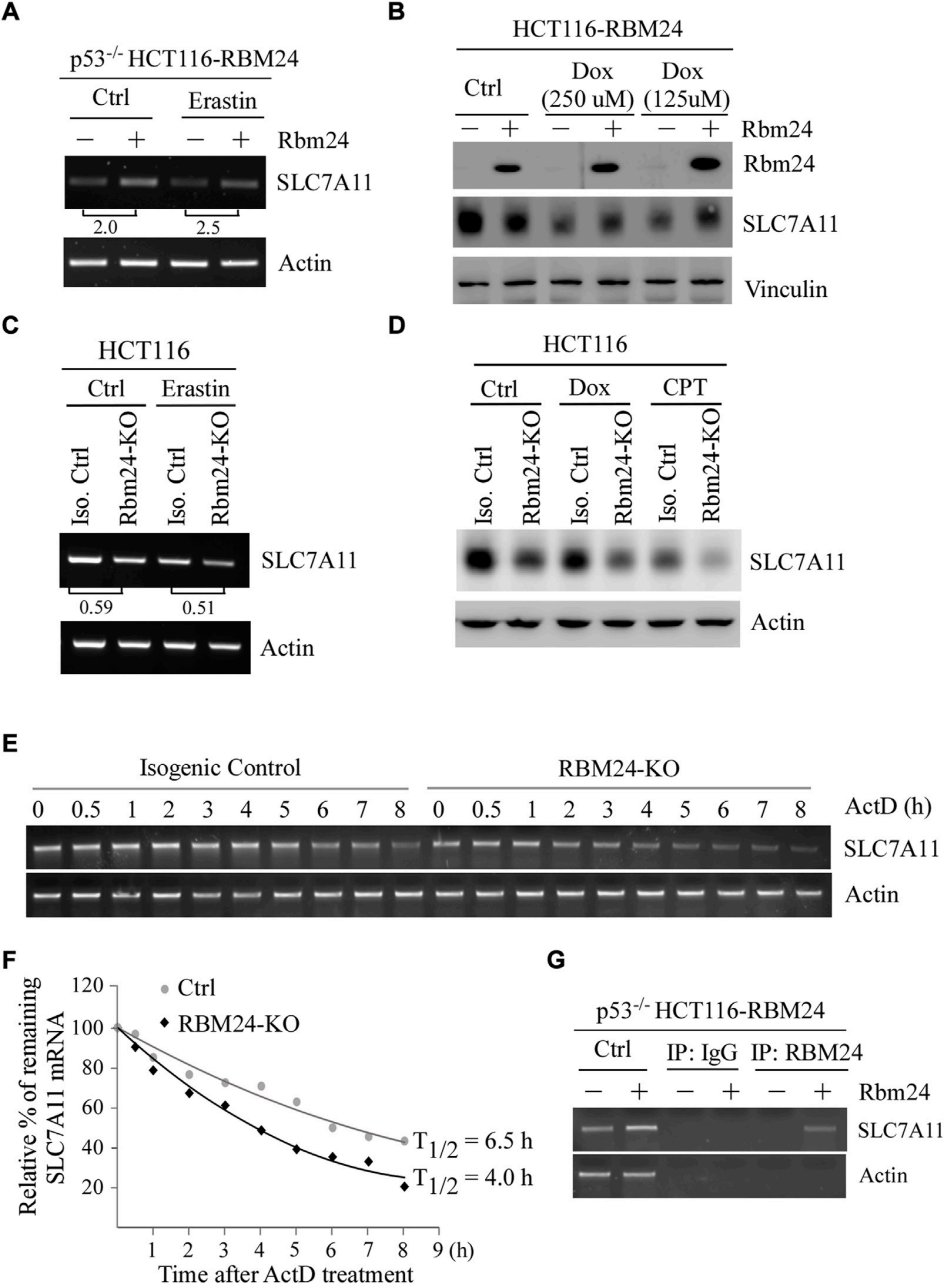
RBM24 inhibits ferroptosis *via* modulating SLC7A11 expression. **(A)** The level of SLC7A11 mRNA was measured in p53^−/−^ HCT116 cells with or without RBM24 expression, followed by mock treatment or treatment with Erastin (40 mM) for 8 h. The relative fold change of SLC7A11 transcript was calculated by dividing the relative abundance of SLC7A11 transcript in RBM24-epxressing cells by the one in control cells. **(B)** HCT116 cells were uninduced or induced to express RBM24 for 24 h, followed by mock or doxorubicin treatment (125 μM or 250 μM) for 12 h. The levels of RBM24, SLC7A11 and actin proteins were measured by western blot analysis. **(C)** Isogenic control and RBM24-KO HCT116 cells were mock-treated or treated with Erastin (40 μM) for 8 h, followed by RT-PCR analysis to measure the levels of SLC7A11 and actin mRNAs. The relative fold change of SLC7A11 transcript was calculated by dividing the relative abundance of SLC7A11 transcript in RBM24-KO cells by the one in control cells. **(D)** Isogenic control and RBM24-KO HCT116 cells were mock-treated or treated with doxorubicin (Dox) or camptothecin (CPT) for 12 h, followed by western blot analysis to measure SLC7A11 and actin proteins. **(E)** Isogenic control and RBM24-KO HCT116 cells were mock-treated or treated with actinomycin D (10 μg/ml) from 0 to 8 h, followed by RT-PCR analysis to measure the levels of SLC7A11 and actin mRNAs. **(F)** Quantification of the relative level of SLC7A11 mRNA in **(E)** normalized to actin by using Visonworks software and densitometry was plotted for the mean ± S.D. **(G)** p53^−/−^ HCT116 cells were uninduced or induced to express RBM24 for 24 h and cell lysate was immunoprecipitated with IgG or anti-RBM24 to bring down the RNA-protein immunocomplex. The binding of RBM24 to SLC7A11 or actin mRNA was visualized by PCR.

Erastin is an inhibitor of SLC7A11 (Stockwell et al., 2017). In addition, several studies have shown that ectopic expression of SLC7A11 inhibits ferroptosis (Jiang et al., 2015; Liu et al., 2019). Thus, to understand the mechanism by which RBM24 inhibits ferroptosis, we tested whether RBM24 directly regulates SLC7A11 expression by using p53^−/−^ HCT116 cells to rule out the potential effect of p53. We found that the level of SLC7A11 transcripts was increased by RBM24 in p53^−/−^ HCT116 cells regardless of Erastin treatment (Figure 4A). In line with this, we found that ectopic RBM24 increased expression of SLC7A11 protein in p53^−/−^ HCT116 cells treated with doxorubicin (Figure 4B). To verify this, isogenic control and RBM24-KO HCT116 cells were used. We found that loss of RBM24 led to decrease in the levels of SLC7A11 transcripts in HCT116 cells with or without Erastin treatment. Similarly, the levels of SLC7A11 proteins were decreased by RBM24-KO in HCT116 cells untreated or treated with camptothecin or doxorubicin (Figure 4D).

RBPs are known to posttranscriptionally modulate their targets (Lukong et al., 2008; Mohibi et al., 2019). Since SLC7A11 mRNA was altered by RBM24 (Figure 4A and Figure 4C), the half-life of SLC7A11 mRNA was measured in isogenic control and RBM24-KO cells mock-treated or treated with Actinomycin D for various times. Actinomycin D is known to inhibit the synthesis of *de novo* mRNAs (Perry and Kelley, 1970) and thereby widely use to measure mRNA stability. We found that the half-life of SLC7A11 mRNA was decreased from 6.5 h in isogenic control cells to 4.0 h in RBM24-KO cells (Figures 4E,F). Moreover, to verify that RBM24 modulates stability of SLC7A11 mRNA, RNA-Chip assay was performed with p53^−/−^ HCT116 cells with or without RBM24 expression. We found that RBM24 was able to directly bind to SLC7A11 mRNA (Figure 4G). As a negative control, RBM24 did not bind to actin mRNA (Figure 4G). Together, these data indicate that SLC7A11 is regulated by RBM24 *via* mRNA stability.

### SLC7A11 is required for RBM24-mediated suppression of ferroptosis

**FIGURE 5:**
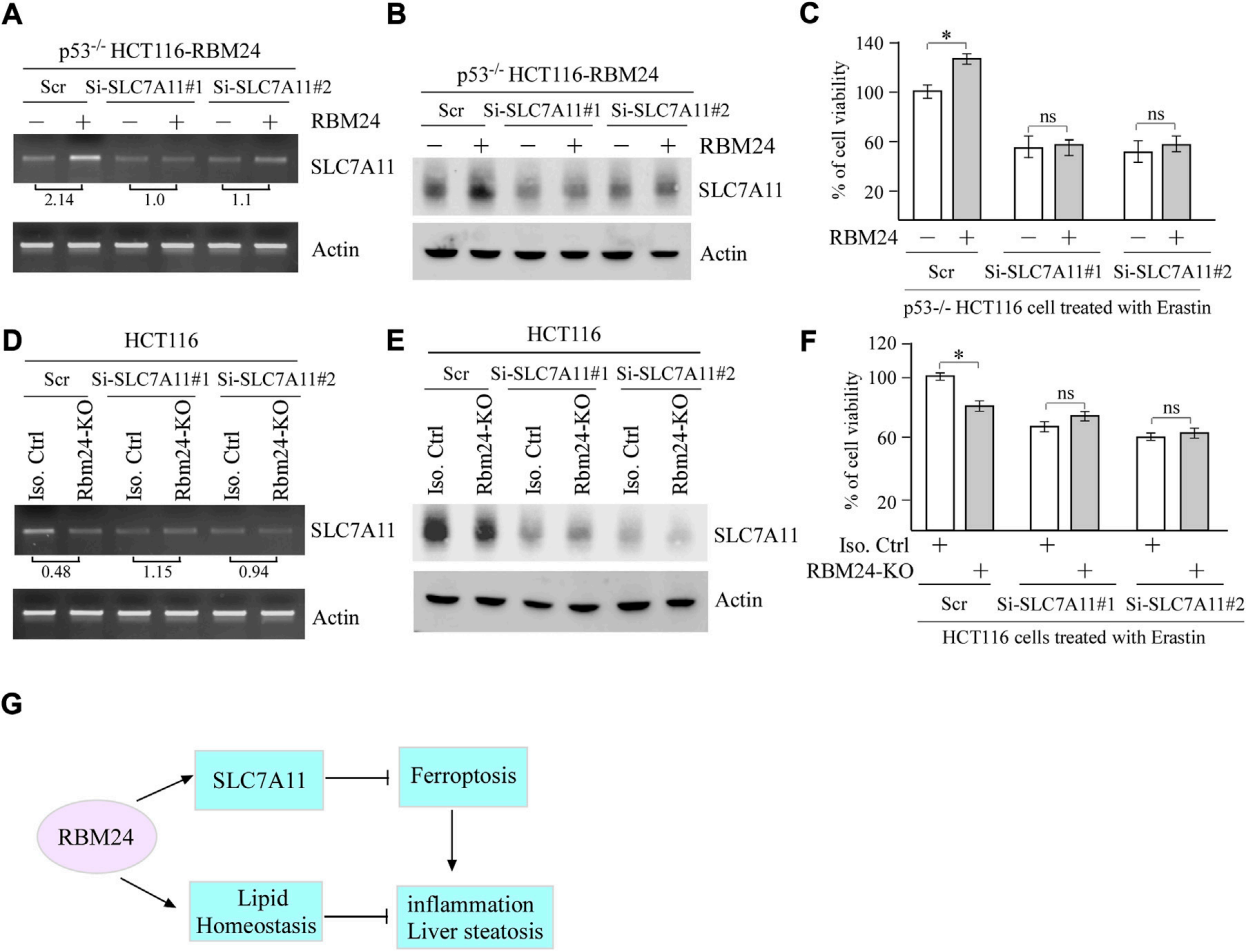
SLC7A11 is required for RBM24-mediated suppression of ferroptosis. **(A,B)** p53^−/−^ HCT116 cells were transiently transfected with a scrambled or SLC7A11 siRNA for 2 days, uninduced or induced to express RBM24 for 24 h, followed by Erastin treatment for 8 h. Both RT-PCR and western blot analyses were performed to measure the levels of SLC7A11 and actin mRNAs **(A)** and proteins **(B)**, respectively. The relative fold change of SLC7A11 transcript was shown below the lane **(C)** p53^−/−^ HCT116 cells were treated as in **(A)**, followed by cell viability assays. The experiments were performed in triplicates **(D,E)** Isogenic control and RBM24-KO HCT116 cells were transiently transfected with a scrambled or SLC7A11 siRNA for 3 days, followed by Erastin treatment for 8 h. The levels of SLC7A11 and actin mRNAs **(D)** and proteins **(E)** were measured by RT-PCR and western blot analyses, respectively. The relative fold change of SLC7A11 transcript was shown below the lane. **(F)** HCT116 cells were treated as in **(D)**, followed by cell viability assay. The experiments were performed in triplicates. **(G)** A proposed model for the role of RBM24 in modulating inflammation and liver steatosis by regulating SLC7A11 and lipid homeostasis.

To determine whether SLC7A11 plays a role in RBM24-mediated suppression of ferroptosis, two SLC7A11 siRNAs were designed and transiently transfected in p53^−/−^ HCT116 cells with or without RBM24 expression along with a scrambled siRNA as a control. As expected, the levels of SLC7A11 mRNAs and proteins were increased by ectopic RBM24, which were decreased by SLC7A11 siRNAs as compared to a scrambled siRNA (Figures 5A,B). Importantly, we found that upon treatment with Erastin, SLC7A11 siRNAs abrogated the protection of ferroptosis mediated by RBM24 (Figure 5C). To verify this, SLC7A11 siRNAs were transiently transfected into isogenic control and RBM24-KO cells followed by treatment with Erastin. As expected, Erastin-induced ferroptosis was inhibited by RBM24-KO or SLC7A11-KD. However, Erastin-induced ferroptosis was not further increased by combined RBM24-KO and SLC7A11-KD (Figures 5D–F). Together, these data indicate that SLC7A11 is a mediator of RBM24 in ferroptosis.

## Discussion

Previous studies have suggested that RBM24 and RBM38 have distinct and overlapped biological functions (Miyamoto et al., 2009; Zhang et al., 2010; Xu et al., 2014; Zhang et al., 2018a; Shao et al., 2020a). In the current study, we found that RBM24-deficiency leads to distinct and more profound alterations in lipid profiles as compared to RBM38-deficiency. We also found that mice deficient in Rbm24 were prone to liver steatosis and chronic inflammation but not spontaneous tumors as compared to Rbm38-deficient mice. To understand the underlying mechanism, we found that RBM24 plays a critical role in protecting cells from Erastin-induced ferroptosis by stabilizing SLC7A11 mRNA. A proposed model for the role of RBM24 in modulating inflammation and liver steatosis were shown in Figure 5G.

Lipidomic analysis indicated that when compared to RBM38-deficiency, RBM24-deficiency showed more profound changes in various lipid classes, including saturated fatty acids, glycerolipids, cholesterol esters, phospholipids, and lysopoholipids (Figure 1). We would like to note that these alterations are consistent with the phenotypes observed in RBM24-deficient mice. For instance, many studies have shown that altered lipid metabolism contributed to chronic inflammation by inducing oxidation of lipids, which would generate excessive lipid mediators (Glass and Olefsky, 2012; Anand, 2020) and thereby promote inflammation (Fritsche, 2015). In addition, liver steatosis is characterized by the accumulation of fat in hepatocytes (Vanni et al., 2010; Shao et al., 2020b) and is clearly associated with increased levels of DAGs and TAGs (Puri et al., 2007). These data indicated that RBM24 plays a role in maintaining proper cellular lipid homeostasis and thereby, prevents organs from pathological impairment. However, several questions still remain. First, RBM24 targets responsible for alterations of various lipid classes remain to be identified. Second, it is unclear how these lipid alterations contribute to the pathological processes mediated by RBM24-deficiency. Addressing these questions would help us further understand the role of RBM24 in lipid metabolism. Interestingly, we also found that RBM38-deficiency, but not RBM24-deficiency, led to marked increase in PC (phosphatidylcholine) (Figure 2E). PC accounts for >50% of the Phospholipids in most eukaryotic membranes. Importantly, PC had been shown to promote cancer cell survival and is frequently increased in many type of cancers (Cheng et al., 2016). Thus, it is possible that RBM38 plays a role in modulating PC biosynthesis through the Kennedy pathway, PEMT pathway and the Lands’ cycle. Thus, further studies are needed to determine the underlying mechanism.

Ferroptosis is characterized by accumulation of lipid oxidation products and associated lipophilic reactive oxygen species (ROS) in cellular membranes (Dixon et al., 2012). In our study, we found that RBM24 protects cells from ferroptosis by stabilizing SLC7A11 mRNA (Figures 3–5). We are aware that RBM24 represses mRNA translation of p53, which is also known to represses SLC7A11 (Jiang et al., 2015). Thus, p53^−/−^ HCT116 cells were used and similar results were obtained as the ones in HCT116 cells (Figure 3A, Figure 4A, and Figures 5A,B). These data indicated that RBM24 regulates SLC7A11 independent of p53. We would like to note that depending on the context, p53 can either promote or inhibit ferroptosis. Since RBM24 is a target of p53, it is possible that RBM24 is a mediator of p53 in suppression of ferroptosis. Moreover, recent studies have shown that ferroptosis plays an important role in initiating inflammation in nonalcoholic steatohepatitis, thereby leading to liver damage (Tsurusaki et al., 2019). Thus, it would be interesting to test whether ferroptosis inhibitors, such as rosiglitazone, an inhibitor of ACSL4 (Doll et al., 2017), and Trolox, an antioxidant vitamin E analog, would alleviate the chronic inflammation and liver steatosis in RBM24-deficient mice.

In this study, we found that RBM24-heterozygous mice were not prone to spontaneous tumors. It is possible that one allele of RBM24 is sufficient to protect mice from tumorigenesis. Indeed, TCGA database showed that RBM24 is down-regulated in several types of cancers, including glioblastoma multiforme (GB) and brain lower grade glioma (LGG). Previous studies have shown that both GB and LGG tumors were resistant to traditional chemotherapy-induced apoptosis (Li et al., 2017). Thus, it would be interesting to determine the role of RBM24 in these tumors, which may lay a foundation for the development of novel cancer therapeutics.

## Data Availability

The original contributions presented in the study are included in the article/Supplementary Material, further inquiries can be directed to the corresponding author.
