# Supplementary material for: The RNA-binding protein RBM24 regulates lipid metabolism and SLC7A11 mRNA stability to modulate ferroptosis and inflammatory response
Source: Front Cell Dev Biol. 2022 Nov 21;10:1008576. doi: 10.3389/fcell.2022.1008576 (PMC9720322; doi:10.3389/fcell.2022.1008576)

Supplemental Table S1: Wild type (WT) mice (n=56) - survival time, tumor spectrum, inflammation, and other abnormalities

ID	Gender	Survival (Wks)	Tumor	Inflammation	Other abnormalities
5	F	134	-	-	-
7	F	117	-	-	-
16	F	100	-	-	-
22	F	109	-	-	-
25	F	109	-	-	-
44	F	90	-	-	-
55	F	104	T-LBL	-	-
64	F	120	-	-	-
2	M	127	-	-	-
3	M	117	-	Liver	-
12	M	127	-	-	-
13	M	127	-	-	-
20	M	122	-	-	-
23	M	122	-	-	-
26	M	127	-	Liver/Salivary gland	-
34	M	124	DLBCL	-	-
37	M	134	-	-	-
62	M	128	-	-	-
45	M	133	-	-	-
49	M	117	-	-	-
50	M	113	T-LBL/ DLBCL	-	-
56	M	117	DLBCL	-	-
59	M	119	DLBCL	-	-
65	M	106	-	-	-
69	M	102	DLBCL	-	Spleen hyperplasia
70	M	103	-	-	Thymus hyperplasia
71	M	90	-	-	-
1-24-2	M	83	-	-	Liver steatosis
2-15-2	F	140	Lymphoma	-	EMH in liver
2-19-6	M	132	-	Pancreas	EMH in Spleen
2-19-2	F	143	-	-	EMH in Spleen
3-11-3	M	129	-	-	-
3-28-5	F	85	-	-	EMH in spleen/Liver teatosis
3-9-7	M	129	-	-	-
5-12-3	F	99	Lymphoma	-	EMH in Spleen
7-9-9	F	116	-	-	-
8-2-6	M	120	-	-	-
10-24-7	F	130	-	Pancreas	EMH in spleen
10-26-6	F	129	Histiocytic sarcoma	-	EMH in spleen/liver
11-10-7	M	121	-	-	-
11-7-3	F	121	-	-	-
11-29-2	F	144	-	-	EMH in spleen/Liver steatosis
12-2-4	F	113	-	-	-
12-20-7	F	115	-	-	EMH in Spleen
11-9-6	F	96	DLBCL	Skin	-
1-19-1	F	86	-	Skin	-
11-10-15	F	105	-	Skin/Pancreas	-
12-25-6	M	111	-	-	Hepatocirrhosis
1-19-5	F	109	Lymphoma	-	-
7-22-4	M	86	-	-	-
7-22-7	M	126	-	Kidney	-
11	M	111	N/A		Found dead
42	M	111	N/A		Found dead
43	M	107	N/A		Found dead
46	M	117	N/A		Found dead
52	M	101	N/A		Found dead

These mice were from published studies (Zhang et al, 2014, PNAS, 111 (52) 18637-18642; Zhang et al, 2017, Genes & Dev, 31:1243-56)

T-LBL: Thymic lymphoblastic lymphoma; DLBCL: Diffuse large B-cell lymphoma; N/A: not applicable; EMH: extramedullary hematopoiesis

Supplemental Table S2: *Rbm38*$^{-/-}$ mice (n=30) - survival time, tumor spectrum, inflammation, and other abnormalities

ID	Gender	Survival (Wks)	Tumor	Inflammation	Other abnormalities
1-12-2	M	100	Lymphoma	Pancreas/Lung/Liver/Salivary gland	EMH in spleen
1-24-5	M	83	-	Lung/Liver	-
1-11-3	M	100	-	Lung	-
1-11-14	F	110	Histiocytic sarcoma	Pancreas/Lung/Liver/Salivary gland/Kidney	EMH in spleen
2-14-2	M	109	-	Lung	EMH in spleen
2-14-3	M	101	-	-	EMH in spleen
2-25-1	F	107	Lymphoma	-	EMH in spleen
3-7-2	F	101	Lymphoma	Lung/Liver	EMH in spleen
3-7-5	M	129	Lymphoma	Kidney/Liver	EMH in liver
3-7-4	M	129	Hepatoma	Pancreas/Kidney/Liver	EMH in spleen
6-15-3	F	90	-	Liver/Lung	EMH in spleen and liver;Liver steatosis
8-2-3	F	94	-	Kidney	EMH in spleen
9-30-4	M	102	Hemangiosarcoma	Pancreas/Lung/Liver	EMH in spleen and liver
10-26-3	F	75	-	Pancreas/Lung	EMH in spleen
11-3-14	M	77	-	-	EMH in spleen
11-10-10	M	124	Lymphoma and hemangiosarcoma	Pancreas/Lung/Kidney/Salivary gland	EMH in spleen and liver
11-16-14	M	128	-	Lung	EMH in spleen and liver
11-16-7	F	125	Hepatoma and lymphoma	Pancreas/Lung/Liver/Salivary gland	EMH in spleen
12-19-3	M	123	Lymphoma	Salivary gland	EMH in spleen
12-19-8	M	118	Hepatoma	Pancreas	EMH in spleen and liver
12-19-6	F	87	-	-	-
12-24-2	M	122	-	Liver	Liver steatosis
12-28-5	F	102	Hepatoma and lymphoma	Pancreas/Lung/Liver/Salivary gland	EMH in spleen
5-13-1+2	M	83	-	Lung/Salivary gland	-
11-26-1	F	100	-	Kidney/Lung/Salivary gland	EMH in spleen, Lymphoid hyperplasia
8-31-6	M	100	-	Kidney;Lung	EMH in spleen
7-16-1	M	83	-	Kidney/Lung/Liver/Salivary gland	EMH in spleen, Liver steatosis
6-17-2	M	95	Lymphoma	Kidney	EMH in spleen
6-9-3	F	96	Lymphoma	Salivary gland	EMH in spleen, Lymphoid hyperplasia
5-13-6	M	101	Hemangioma	Kidney/Lung/liver/Salivary gland	EMH in spleen, Steatosis

The data of the first 23 mice were from published studies (Zhang et al, 2014, PNAS, 111 (52) 18637-18642)
EMH: extramedullary hematopoiesis

Supplemental Table S3: ***Rbm24***$^{+/-}$ **mice (n=22) - survival time, tumor spectrum, steatosis, inflammation, and other abnormalities**

ID#	Gender	Survival (Wks)	Tumor	Steatosis	Inflammation	Other abnormalities
5-30-1	F	120	-	-	Liver/Salivary gland/ kidney/ pancreas/lung	EMH
6-24-3	M	90	Adenocarcinoma	-	Liver/lung/ Salivary gland/GI Tract	EMH/ Liver fibrosis
12-31-5	M	102	Hemangioma	-	Liver/pancreas/kidney/salivary gland	EMH
12-19-3	M	53	-	Y	Lung/kidney/salivary gland/liver/GI tract	EMH
7-14-3	F	114	-	Y	Liver/lung/GI tract	EMH
5-24-5	M	120	-	Y	Liver/lung/GI tract	EMH
6-5-4	F	118	-	Y	Liver/Kidney/Salivary gland/lung	-
12-19-5	M	62	-	-	Liver/salivary gland	EMH
7-21-6	M	77	-	Y	Liver/Salivary gland/Kidney	-
5-1-4	M	139	-	-	Salivary gland	EMH
6-24-1	F	114	-	Y	Lung/Salivary gland/Fat/Kidney	EMH
11-14-1	F	79	sarcoma	-	Kidney/Salivary gland/lung	EMH
5-7-3	F	113	sarcoma	-	Kidney/Liver/Lung	EMH
5-24-3	M	104	-	Y	Kidney/salivary gland	EMH
5-24-4	M	104	-	-	Salivary gland	EMH
5-4-9	M	50	-	Y	Kidney/pancreas/salivary gland	EMH
6-1-3	F	93	-	Y	Kidney/Liver/salivary gland	EMH
6-5-1	F	93	Lymphoma	-	salivary gland	SMH
12-23-1	M	105	-	-	Liver/Fat/Salivary gland/Kidney/lung	EMH
12-23-2	M	104	-	-	Salivary gland/Kidney	EMH
12-19-4	F	53	-	-	Liver/Lung/salivary gland	EMH
6-1-7	M	98	-	-	Liver/Kidney/Salivary gland	EMH

EMH: Extramedullary Hematopoiesis; SH: Splenic Hyperplasia

Supplemental Figure 1

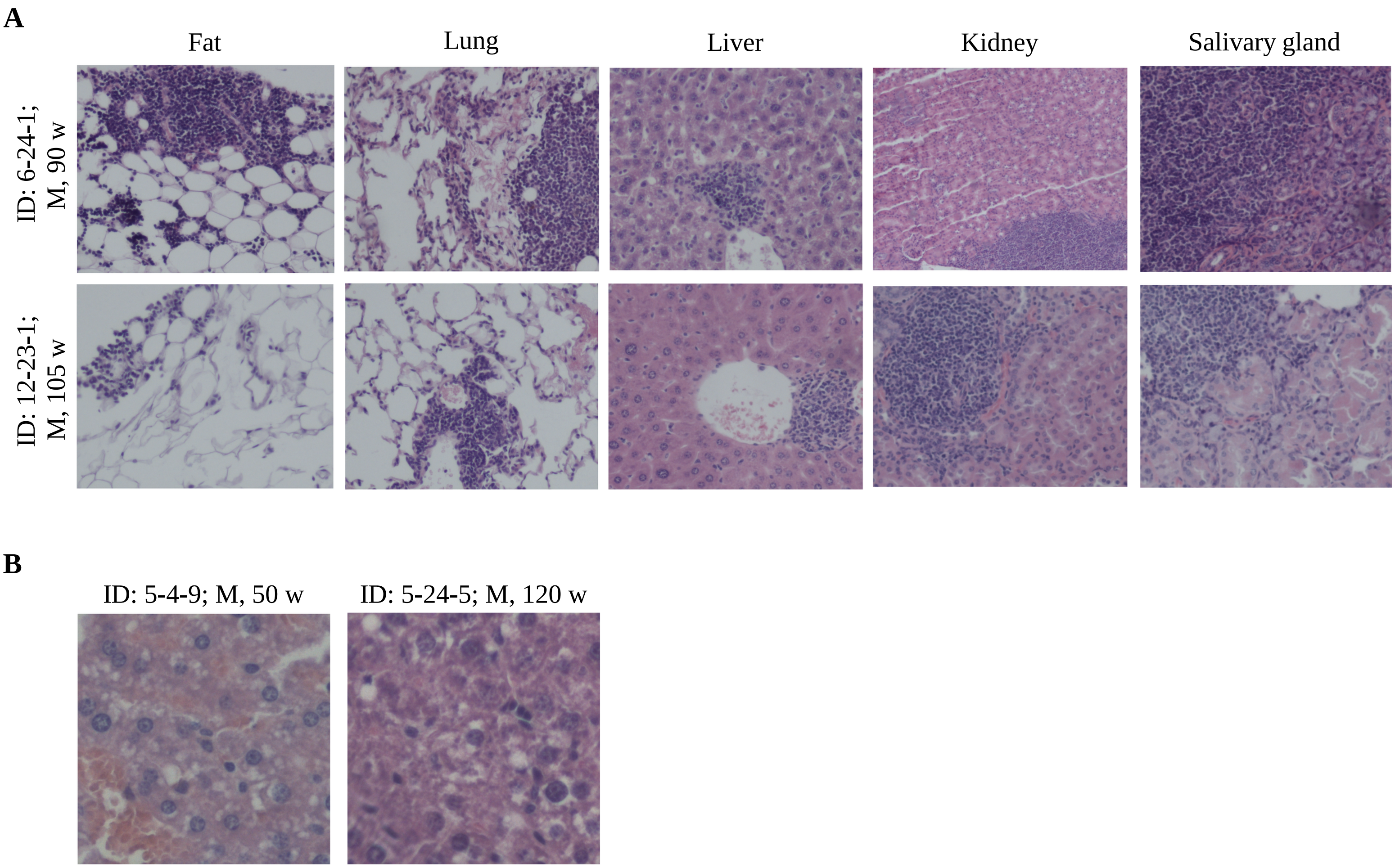

Supplement: Supplementary file 1 [file DataSheet1.PDF]
